# Differential Effects of Oleoyl Serine and HU-910 on Anxiety-like and Depression-like Behaviors in Male and Female WKY Rats

**DOI:** 10.3390/ijms27073177

**Published:** 2026-03-31

**Authors:** Jenna Gellman, Natalia Zemliana, Yoni Loterstein, Elin Kachuki Dory, Devorah Matas, Gal Shoval, Eyal Sharon, Igor Koman, Gil Zalsman, Lee Koren, Aron Weller, Natalya M. Kogan

**Affiliations:** 1 Gonda Brain Research Center, Bar-Ilan University, Ramat Gan 5290002, Israel; gellmanjenna@gmail.com (J.G.); yloterstein@gmail.com (Y.L.); oeyibgyi@gmail.com (E.K.D.); lee.koren@biu.ac.il (L.K.); 2 Institute for Personalized and Translational Medicine, Department of Molecular Biology, Adelson School of Medicine, Ariel University, Ariel 4070000, Israel; nataliaz@ariel.ac.il (N.Z.); eyalsh@ariel.ac.il (E.S.); igorko@ariel.ac.il (I.K.); 3 Psychology Department, Bar-Ilan University, Ramat Gan 5290002, Israel; 4 Faculty of Life Sciences, Bar Ilan University, Ramat-Gan 5290002, Israel; devorahbio@gmail.com; 5 Geha Mental Health Center, Petah Tiqva 49100, Israel; shovgal@tauex.tau.ac.il (G.S.); zalsman@tauex.tau.ac.il (G.Z.); 6 Gray Faculty for Medical and Health Science, Tel Aviv University, Tel Aviv 6997801, Israel; 7 Division of Molecular Imaging and Neuropathology, Department of Psychiatry, Columbia University, New York, NY 10027, USA

**Keywords:** animal models of depression, Wistar-Kyoto rats, endocannabinoid system

## Abstract

The role of the endocannabinoid system (ECS) in the development of depression and anxiety is being actively studied, with evidence suggesting that elevation of ECS signaling can have anxiolytic and antidepressant properties. The current study explored the therapeutic potential of Oleoyl Serine (OS), an endocannabinoid-like lipid, and HU-910, a synthetic selective Cannabinoid type 2 (CB2) receptors agonist, in depression and anxiety, using both sexes of the depressive-like genetic model: Wistar Kyoto (WKY) rats. The aim was to investigate behavioral and molecular mechanisms associated with acute and sub-chronic intraperitoneal administration of these compounds. We showed that, in females, acutely administered OS yielded antidepressant-like and anxiolytic-like effects in the Forced Swim Test (FST) and Open Field Test (OFT), respectively. In males, OS yielded acute and sub-chronic anxiolytic-like effects. HU-910 yielded an acute anxiolytic-like effect in females and an acute antidepressant-like effect in males. Sub-chronic administration of imipramine (IMI), used as a positive control, yielded an antidepressant-like effect in both sexes but an anxiogenic-like effect in females. Sub-chronic administration of all the treatments increased hippocampal *Cannabinoid Receptor 1* (*CNR1*) mRNA expression (but not *Fatty Acid Amide Hydrolase* (*FAAH*)) in males. Exploratory in silico absorption, distribution, metabolism, and excretion (ADME) profiling suggests that sex-dependent pharmacokinetic variability may partly underlie the observed behavioral differences, in addition to possible pharmacodynamic factors. Our study provides a lead towards unraveling the putative sex differences in response to both conventional antidepressants (e.g., IMI) and emerging pharmacological agents (e.g., OS, HU-910). Further, our study helps advance the field of neuropharmacology by elucidating the anxiolytic-like and antidepressant-like effects of OS and HU-910.

## 1. Introduction

Depression and anxiety are highly comorbid and frequently treatment-resistant disorders. Although Selective Serotonin Reuptake Inhibitors (SSRIs) are the most common pharmacological treatment, a substantial proportion of patients fail to achieve remission or experience adverse side effects [1,2,3,4,5]. Compounding these issues, women are more than twice as likely to experience both disorders, yet sex differences in underlying mechanisms and treatment remain insufficiently understood [6,7,8,9]. Thus, it is of paramount importance to develop mechanistically novel and sex-sensitive therapeutic strategies.

The endocannabinoid system (ECS) appears to be an interesting target for new antidepressant and anxiolytic drugs, as there is increasing evidence of its etiological link to both disorders. The ECS plays a critical role in the regulation of mood and emotional processing, and its modulation may help mitigate symptoms of depression and anxiety [10,11,12,13]. It has been suggested that ECS deficits may contribute to depressive and anxiety-inducing effects on behavior, whereas enhancing ECS signaling could produce antidepressant and anxiolytic effects [14,15], with endogenous cannabinoids such as anandamide (AEA) and 2-arachidonoylglycerol (2-AG) demonstrating anxiolytic and antidepressant-like effects in animal models [16,17,18,19]. However, both compounds face major obstacles as drug candidates—they are psychotropic, act primarily through Cannabinoid type 1 (CB1) receptors, and are chemically unstable, due to their arachidonic acid-based structure, which is prone to oxidation, heat degradation, and photolysis [20,21]. Moreover, both these compounds are oils, which complicates pharmaceutical development. Continuing on from our previous work on the antidepressant-like properties of cannabidiolic acid methyl ester, a stable methylated non-psychotropic derivative of cannabidiolic acid [22,23,24], we sought to explore other non-psychotropic, ECS-related compounds that avoid CB1 activation and offer greater chemical stability and drug-like properties. Oleoyl serine (OS) and HU-910 meet these criteria.

OS is an endogenous N-acyl amide that, while not binding directly to CB1 or CB2 receptors, is structurally related to ECS lipids and may interact with orphan G-protein coupled receptors or lipid-sensitive signaling pathways involved in mood regulation [25]. Related non-psychotropic lipid mediators, including oleoyl ethanolamide (OEA) and oleoyl glycine (OG), have demonstrated antidepressant- and anxiolytic-like effects in preclinical models. OEA, an analog of anandamide, exerts its antidepressant-like effects by normalizing brain-derived neurotrophic factor (BDNF) levels and reducing oxidative stress in models of chronic stress [26]. OG significantly altered the levels of other endocannabinoids and N-acylethanolamines in the brain, particularly in the prefrontal cortex and hypothalamus in mild Traumatic Brain Injury models. Further, OG improved motor function and reduced anxiety-like behaviors [27]. Despite these findings, the behavioral and affective effects of OS in depression- and anxiety-related models remain largely unexplored.

HU-910 is a synthetic CB2-selective agonist explicitly designed to avoid CB1-mediated psychoactivity while retaining the anti-inflammatory and neuroprotective effects of cannabinoids [28]. Since the majority of CB2 receptors are located in the periphery, such as in immune cells [29,30,31], their activation by CB2 receptor agonists can moderate inflammation and immune responses [32]. Given the growing evidence linking depression and anxiety to immune dysregulation and neuroinflammation [33,34,35,36,37], Cannabinoid type 2 (CB2) receptor agonists may exert antidepressant- and anxiolytic-like effects via immunomodulatory pathways [38,39,40,41]. Although HU-910 has demonstrated behavioral efficacy in other neuropsychiatric models such as schizophrenia [28], its effects in models of depression and anxiety have yet to be elucidated.

Importantly, both OS and HU-910 are solid at room temperature, chemically stable, and have shown anti-inflammatory effects, suggesting they may modulate mood-related inflammation—a growing focus in depression and anxiety research [42,43]. To evaluate these compounds, we utilized the Wistar Kyoto (WKY) rat, a well-characterized genetic model displaying robust depressive-like and anxiety-like phenotypes [44,45,46,47,48,49,50], as well as resistance to conventional antidepressant treatments [44,50]. Importantly, sex differences have been reported in this strain [47,50], making it particularly suitable for investigating potential sex-specific drug responses.

Using both male and female WKY rats, the present study investigated the behavioral and molecular effects of OS and HU-910 following acute and sub-chronic (two-week) administration. By targeting non-psychotropic ECS-related mechanisms and including biological sex as a variable, this study aimed to evaluate the therapeutic potential and sex-dependent effects of these compounds in a treatment-resistant model of depression and anxiety.

## 2. Results

### 2.1. Acute Experiment Results

#### 2.1.1. HU-910 and OS Induced Differential Acute Antidepressant-like Effects in Male and Female WKY Rats in the Forced Swim Test (FST)

Male and female groups responded differently to acute treatment in the FST for depression-like behavior.

**Figure 1:**
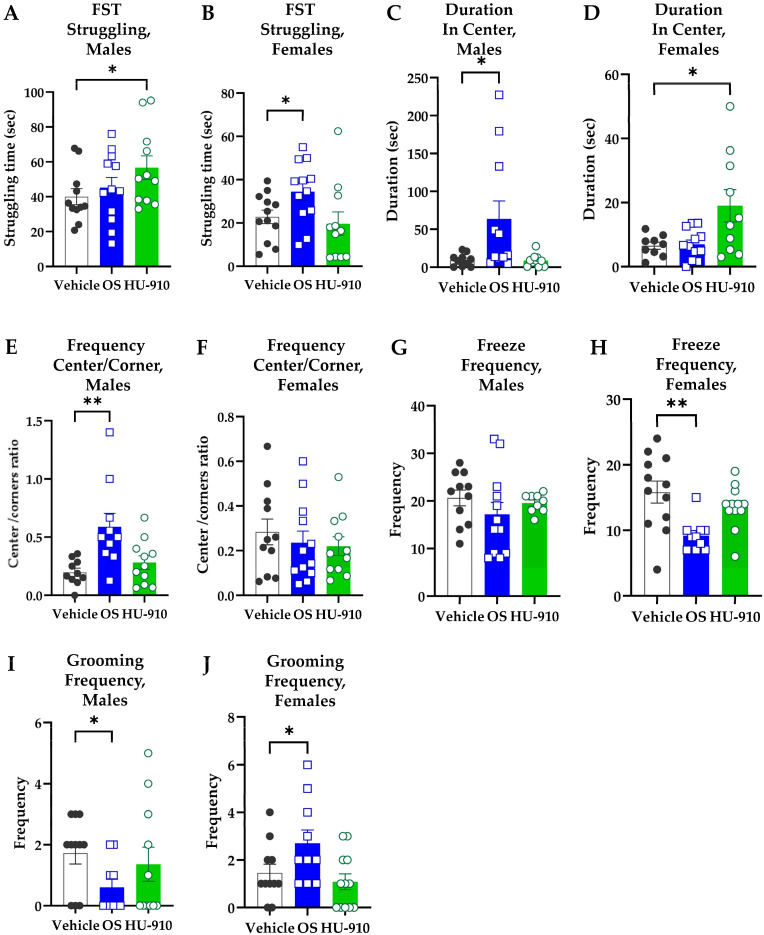
**Behavioral tests for WKY rats after acute treatment.** Duration of struggling in the Forced Swim Test (FST) of males (**A**) and females (**B**). Duration in the Open Field TEST (OFT) Center for males (**C**) and females (**D**). Frequency ratio in the OFT Center/corner entries for males (**E**) and females (**F**). Freeze frequency in the OFT for males (**G**) and females (**H**). Grooming frequency in the OFT for males (**I**) and females (**J**). The rats (n = 11–12 per group) received a single i.p. injection of vehicle, 7.5 mg/kg Oleoyl Serine (OS), or 5 mg/kg HU-910. The data are presented as mean ± SEM. * *p* < 0.05, ** *p* < 0.01. The exact statistical test used in each experiment is described in Section 4.

In males, HU-910 treatment increased struggling duration (Figure 1A), indicating a reduced manifestation of “behavioral despair”. Since the data were not normally distributed, they were analyzed by a Kruskal–Wallis Analysis of Variance (ANOVA). Neither the ANOVA result (*p* = 0.1601) nor Dunn’s post hoc comparison correction of vehicle vs. HU-910 groups (*p* = 0.0581) was significant. However, a direct two-tailed Mann–Whitney U-test between these two groups indicated a statistically significant increase in struggling duration following HU-910 administration (*p* = 0.0349).

In females, Oleoyl Serine increased struggling duration (Figure 1B), indicating a reduced manifestation of “behavioral despair”. The SDs were not different, and the distribution was normal; thus, the data were analyzed by a regular one-way ANOVA, which revealed a significant overall difference among groups (*p* = 0.0481), followed by Bonferroni post hoc correction for multiple comparisons, which appeared insignificant (*p* = 0.0594 for vehicle vs. OS group comparison). However, a direct two-tailed t-test between these two groups indicated a statistically significant increase in struggling duration following OS administration (*p* = 0.0350).

#### 2.1.2. HU-910 and OS Induced Differential Acute Anxiolytic-like Effects in Male and Female WKY Rats in the Open Field Test (OFT)

Male and female groups responded differently to acute treatment in the OFT for anxiety-like behavior.

In males, OS produced an anxiolytic-like effect, demonstrated by OS-treated males spending more time in the central part of the arena (see Figure 1C) and making significantly more entries to the center compared to the vehicle-treated animals (Figure 1E). Regarding duration in the OFT center (Figure 1C), the Brown–Forsythe test for SDs was significant (*p* = 0.0330). The data of both vehicle and HU-910 groups were not normally distributed; thus, the data were analyzed by a Kruskal–Wallis ANOVA (*p* = 0.0243), followed by Dunn’s post hoc multiple comparisons correction (*p* = 0.0440 for vehicle vs. OS group comparison), showing a significant difference between the groups. Regarding entries into the OFT center, the Brown–Forsythe test for SDs was significant (*p* = 0.0031), and the distribution was normal; thus, the data were analyzed by a Brown–Forsythe ANOVA (*p* = 0.007), followed by Dunnett T3 post hoc multiple comparisons correction (*p* = 0.0079 for vehicle vs. Figure 1E). OS group comparison showed that OS significantly elevates the frequency of entrances to the center of the arena.

In females, HU-910 produced an anxiolytic-like effect, demonstrated by HU-910-treated females spending significantly more time in the center compared to vehicle-treated females (Figure 1D). Regarding duration in the OFT center, the Brown–Forsythe test for SDs was significant (*p* = 0.0036), and the distribution was normal. Thus, the data were analyzed by a Brown–Forsythe ANOVA (*p* = 0.0240), followed by Dunnett T3 post hoc multiple comparisons correction (*p* = 0.0355 for vehicle vs. HU-910 in group comparison). However, there was no significant difference in the center/corner entering frequency ratio (Figure 1F). Regarding entries into the OFT center (Figure 1F), the SDs were not different, and the distribution was normal; thus, a regular one-way ANOVA was conducted, and it gave an insignificant result (*p* = 0.6642).

#### 2.1.3. Acute OS Treatment Altered Stress Coping Behaviors in the OFT

In addition to spatial exploration measures, OS treatment also affected behaviors associated with stress coping in the OFT.

In females, OS led to more stable behavior, reflected by a significant reduction in Freeze Frequency (Figure 1H). Since the Brown–Forsythe test for SDs was significant (*p* = 0.0334) and the distribution was not normal, the data were analyzed by a Kruskal–Wallis ANOVA (*p* = 0.0032), followed by Dunn’s post hoc multiple comparisons correction (*p* = 0.0018 for vehicle vs. OS group comparison). A similar tendency was observed in the OS-treated males; however, the result was not significant (Figure 1G). Since the Brown–Forsythe test for SDs was significant (*p* = 0.0211), the data were analyzed by a Brown–Forsythe ANOVA, which gave an insignificant result (*p* = 0.3756).

OS also produced sex-dependent effects on grooming frequency. In females, OS resulted in increased grooming (Figure 1J), whereas in males, OS resulted in reduced grooming (Figure 1I). For groom frequency in females, since the SDs were not different and the distribution was normal, the data were analyzed by a regular one-way ANOVA (*p* = 0.0299), followed by Bonferroni post hoc correction for multiple comparisons (*p* = 0.0487 for vehicle vs. OS group comparison). For groom frequency in males, since the SDs were not different, and the distribution was not normal, a Kruskal–Wallis ANOVA was conducted, and it did not reach significance (*p* = 0.1496). Dunn’s comparison of vehicle vs. OS was also insignificant (*p* = 0.1032). However, a direct two-tailed Mann–Whitney U-test between these two groups indicated a statistically significant difference (*p* = 0.0366). While induced grooming may usually be an indication of successful stress coping and reduced anxiety, the precise interpretation must be made by considering all the other test results.

### 2.2. Sub-Chronic Experiment Results

#### 2.2.1. HU-910 and OS Did Not Show Sub-Chronic Antidepressant-like Effects in WKY Rats in the Forced Swim Test (FST)

**Figure 2:**
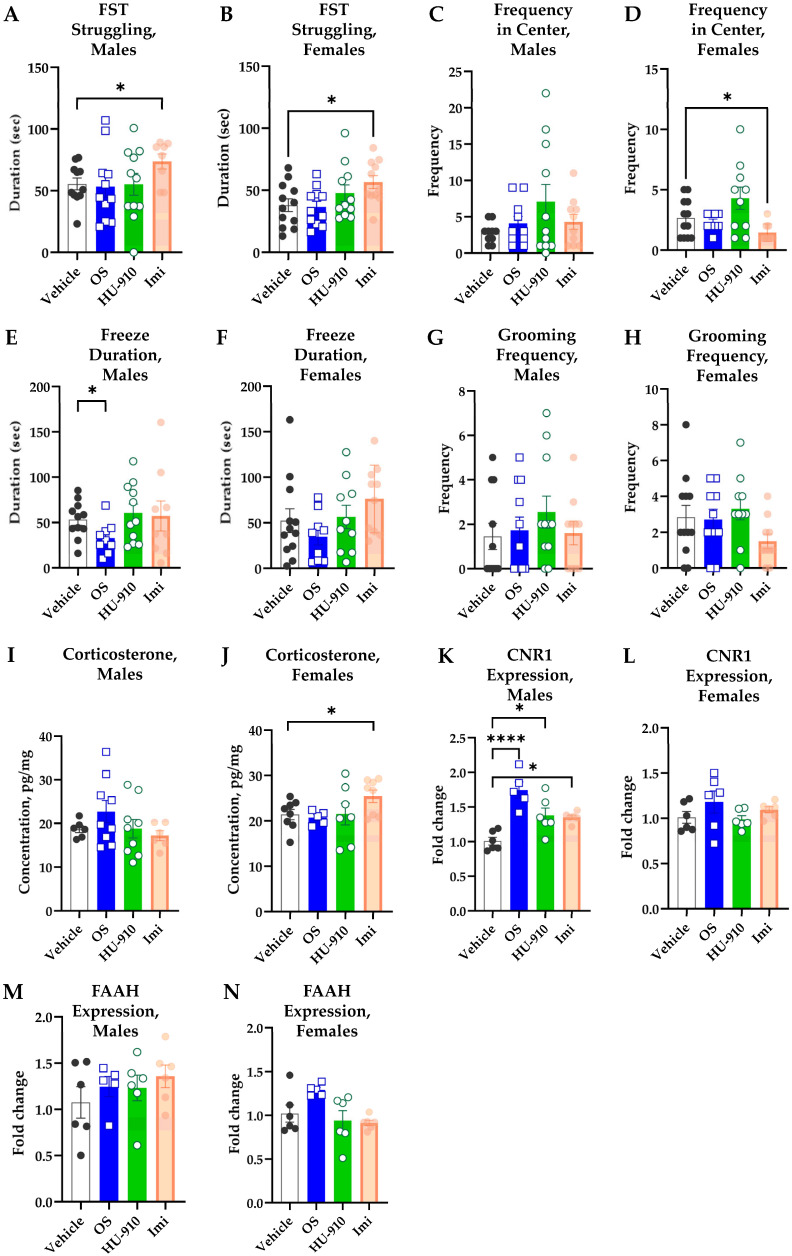
**Behavioral and Molecular tests for WKY rats after sub-chronic treatment.** Duration of struggling in the FST of males (**A**) and females (**B**). Frequency in the OFT Center for males (**C**) and females (**D**). Freeze duration in the OFT for males (**E**) and females (**F**). Grooming frequency in the OFT for males (**G**) and females (**H**). Hair corticosterone concentrations for males (**I**) and females (**J**). Hippocampal *CNR1* expression for males (**K**) and females (**L**). Hippocampal *FAAH* expression for males (**M**) and females (**N**). The rats (n = 6–12 per group) received a daily i.p. injection of vehicle, 7.5 mg/kg OS, 5 mg/kg HU-910, or 10 mg/kg imipramine (IMI) for 2 weeks. The data are presented as mean ± SEM, and they were normalized by *GAPDH* housekeeping gene expression for *CNR1* and *FAAH* expression via RT-qPCR on hippocampal tissue. * *p* < 0.05, **** *p* < 0.0001. The exact statistical test used in each experiment is described in Section 4.

For the sub-chronic FST study, however, significant differences were noted only for the positive control imipramine (IMI) group, for both males (Figure 2A) and females (Figure 2B). The behavior of the vehicle and treatment groups did not differ. For males, since the SDs were not different and the distribution in the IMI group was not normal, the data were analyzed by a Kruskal–Wallis ANOVA; however, neither the ANOVA result (*p* = 0.1239) nor Dunn’s correction for multiple comparisons (*p* = 0.0746) was significant. A direct two-tailed Mann–Whitney U-test between the vehicle and IMI groups, however, indicated a statistically significant difference (*p* = 0.0201). For females, since the SDs were not different, and the distribution was normal, the data were analyzed by a regular one-way ANOVA (*p* = 0.0438), followed by Bonferroni post hoc multiple comparisons correction, which appeared significant (*p* = 0.0184 for vehicle vs. IMI group comparison).

#### 2.2.2. HU-910 and OS Induced Differential Sub-Chronic Anxiolytic-like Effects in Male and Female WKY Rats in the Open Field Test (OFT)

Although a trend for a positive anxiolytic-like effect, expressed as frequency in the center of the Open Field, exists in the HU-910-treated group, it was not statistically significant for both males (Figure 2C) and females (Figure 2D). For males, since the Brown–Forsythe test for SDs was significant (*p* = 0.0237) and the distribution of the HU-910 group was not normal, the data were analyzed by a Kruskal–Wallis ANOVA; however, neither the ANOVA result (*p* = 0.8857) nor Dunn’s correction of any comparison was significant. For females, since the Brown–Forsythe test for SDs was significant (*p* = 0.0007), the data were analyzed by a Brown–Forsythe ANOVA (*p* = 0.0158), followed by Dunnett T3 correction for multiple comparisons, which appeared significant for vehicle vs. IMI (*p* = 0.0293) but not for vehicle vs. HU-910 (*p* = 0.1342).

Interestingly, the antidepressant IMI had some anxiogenic effect in females, causing fewer entries to the center (Figure 2D). This finding is supported by the (albeit insignificant) trend for more freezing (Figure 2F) and elevated corticosterone levels (Figure 2J), observed in the same animals. For freeze duration in females (Figure 2F), since the SDs were not different and the distribution was normal, the data were analyzed by a regular one-way ANOVA; however, neither the ANOVA result (*p* = 0.1518) nor Bonferroni post hoc correction for multiple comparisons for any group was significant. For corticosterone levels in females (Figure 2J), since the SDs were not different and the distribution was normal, the data were analyzed by a regular one-way ANOVA; however, neither the ANOVA result (*p* = 0.1575) nor Bonferroni multiple comparisons was significant (*p* = 0.0686 for vehicle vs. IMI group comparison). However, a direct two-tailed t-test between the vehicle and IMI groups indicated a statistically significant difference (*p* = 0.0416).

As for freeze duration in males (Figure 2E), OS significantly reduces the freezing duration. Since the SDs were not different and the distribution was normal, a regular one-way ANOVA was conducted. Neither the ANOVA result (*p* = 0.2254) nor Bonferroni post hoc correction for multiple comparisons was significant (*p* = 0.1664 for vehicle vs. OS comparison). However, a direct two-tailed t-test between these two groups indicated a statistically significant difference (*p* = 0.0294). Regarding corticosterone levels in males (Figure 2I), since the SDs were not different and the distribution was normal, a regular one-way ANOVA was conducted. However, neither the ANOVA result (*p* = 0.2805) nor Bonferroni post hoc correction for multiple comparisons was significant.

As for the grooming frequency, it did not differ significantly between the groups for both males and females. For males (Figure 2G), the SDs were not different, and the distribution was not normal; thus, a Kruskal–Wallis ANOVA was conducted. However, neither the Kruskal–Wallis ANOVA result (*p* = 0.5773) nor Dunn’s post hoc correction for multiple comparisons for any group was significant. For females (Figure 2H), the SDs were not different, and the distribution was normal; thus, the data were analyzed by a regular one-way ANOVA. However, neither the ANOVA result (*p* = 0.1943) nor Bonferroni post hoc correction was significant.

In light of these findings, the only meaningful anxiolytic-like result for the treatment groups is a significantly lower freeze duration in OS-treated males (Figure 2E).

#### 2.2.3. HU-910 and OS Induced *Cannabinoid Receptor 1* (*CNR1*) Expression in Male WKY Rat Hippocampi

Following sub-chronic administration of vehicle, OS, HU-910, or IMI, hippocampal expression of ECS-related transcripts was quantified by real-time RT-qPCR. In males, OS, HU-910, and IMI treatments were associated with increased hippocampal *CNR1* expression relative to the vehicle, with the OS effect being the most prominent (Figure 2K). Since the SDs were not different and the distribution was normal, the data were analyzed by a regular one-way ANOVA, which revealed a significant overall difference among groups (*p* < 0.0001), followed by Bonferroni post hoc correction for multiple comparisons, which appeared significant (*p* < 0.0001 for vehicle vs. OS group comparison, *p* = 0.0240 for vehicle vs. HU-910 group comparison, and *p* = 0.0413 for vehicle vs. IMI group comparison). The expression of fatty acid amide hydrolase (FAAH) in males did not change (Figure 2M). Since the SDs were not different and the distribution was normal, the data were analyzed by a regular one-way ANOVA. However, neither the ANOVA result (*p* = 0.3696) nor Bonferroni multiple comparisons was significant.

In females, no statistically significant treatment-related changes were detected for these transcripts. For *CNR1* expression (Figure 2L), since the SDs were not different and the distribution was normal, the data were analyzed by a regular one-way ANOVA. However, neither the ANOVA result (*p* = 0.2725) nor Bonferroni multiple comparisons was significant. For *FAAH* expression (Figure 2N), since the Brown–Forsythe test for SDs was significant (*p* = 0.0318), the data were analyzed by a Brown–Forsythe ANOVA (*p* = 0.0288), followed by Dunnett T3 correction for multiple comparisons, which appeared significant only for the OS vs. IMI group comparison (*p* < 0.001), with a slight trend involving OS elevating *FAAH* expression (*p* = 0.1720 for vehicle vs. OS group comparison).

In both males and females, hippocampal *Cannabinoid Receptor 2* (*CNR2*) mRNA was below the assay’s detection limit under the present experimental conditions.

### 2.3. In Silico ADME Profiling

**Figure 3:**
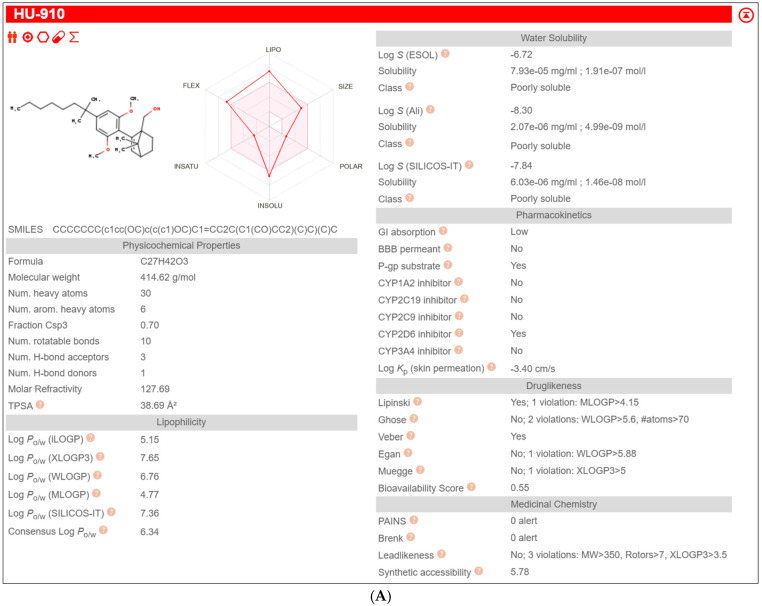

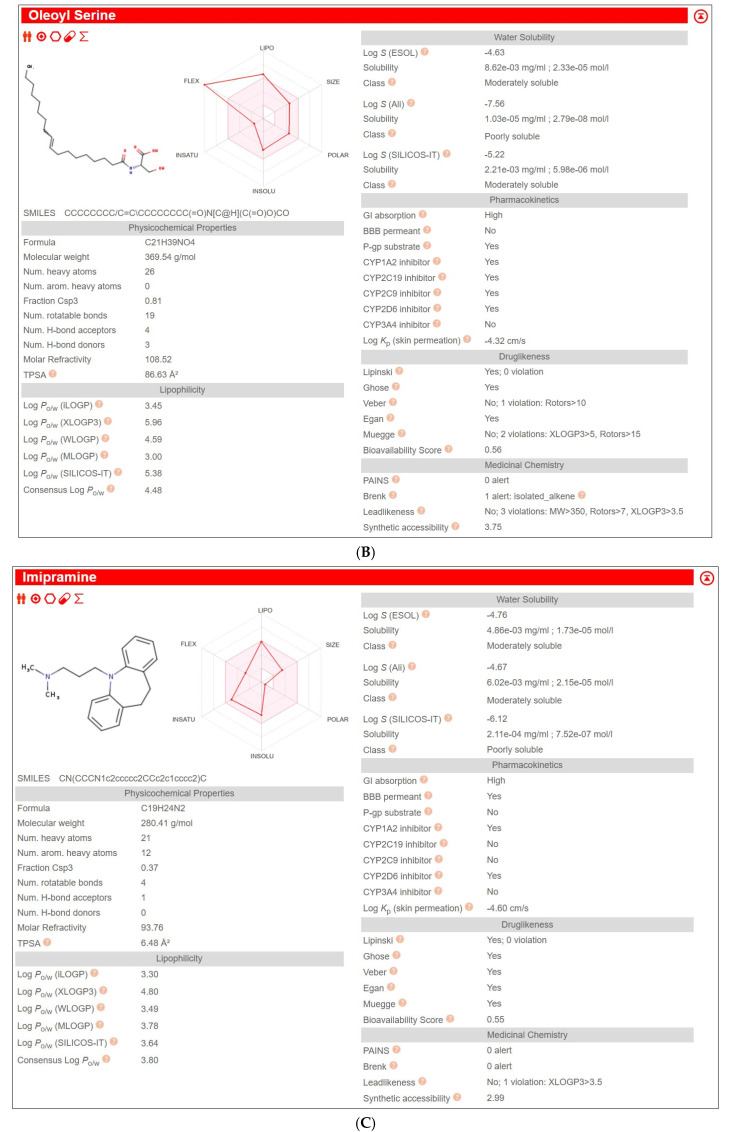
**In silico ADME profiles of the tested compounds generated using SwissADME.** Representative SwissADME output panels for the three compounds used in the present study: (**A**) HU-910, (**B**) oleoyl serine, and (**C**) IMI. The panels summarize predicted physicochemical properties, lipophilicity, aqueous solubility, pharmacokinetic parameters, drug-likeness filters, and medicinal chemistry alerts for each compound. Abbreviations: ADME, absorption, distribution, metabolism, and excretion; BBB, blood–brain barrier; GI, gastrointestinal; P-gp, P-glycoprotein; CYP, cytochrome P450.

Exploratory SwissADME profiling indicates marked differences in predicted disposition properties among the tested compounds: IMI was predicted to be Blood-Brain Barrier (BBB)-permeant and not a P-gp substrate, whereas OS and HU-910 were predicted not to be BBB-permeant and to be P-gp substrates; HU-910 additionally showed extreme lipophilicity and poor predicted solubility (Table 1, Figure 3). These predictions are sex-independent; thus, they do not, by themselves, demonstrate male–female pharmacokinetic differences, but they do identify plausible pathways through which such differences could arise.

## 3. Discussion

### 3.1. Overview of Behavioral and Molecular Findings

Our findings suggest distinct sex- and time-dependent behavioral and molecular effects of OS and HU-910 in WKY rats. At the behavioral level, OS exhibited acute antidepressant- and anxiolytic-like effects in females, evidenced by increased struggling in the FST and reduced freezing, alongside increased grooming, in the OFT. OS produced acute anxiolytic-like effects in males, shown by increased time and entries in the center of the OFT, though grooming behavior decreased. Sub-chronic OS treatment in males reduced freezing, suggesting sustained anxiolytic-like effects, though no antidepressant-like activity was detected at either time point. Acute HU-910 produced antidepressant-like effects selectively in males, indicated by increased struggling in the FST, while sub-chronic treatment was associated with a non-significant trend toward anxiolytic-like behavior. In females, HU-910’s effects were limited to acute anxiolysis, indicated by more time spent in the OFT center, while sub-chronic treatment did not elicit significant changes. These patterns contrast with the positive control, IMI, which induced antidepressant-like effects in both sexes after sub-chronic administration but failed to produce anxiolysis and instead induced anxiogenic-like responses only in females. Taken together, the data highlight compound-specific, sex-dependent, and time-dependent differences in the regulation of affective behaviors.

At the molecular level, sub-chronic administration of all three compounds significantly upregulated hippocampal *CNR1* geneexpression, with OS producing the strongest increase. In contrast, hippocampal *CNR2* transcripts were below the detection limit, and *FAAH* (the main endocannabinoid-metabolizing enzyme) expression was unchanged. These transcriptional adaptations were largely absent in females, mirroring the attenuated or different behavioral responses observed in that sex. Taken together, these findings may reflect sex-dependent differences in ECS recruitment and hormonal modulation. However, pharmacokinetic factors (e.g., sex-dependent differences in drug absorption, distribution, and metabolism) may also contribute to the different behavioral responses to OS and HU-910.

### 3.2. CB1 Recruitment as a Potential Convergent Adaptive Mechanism

The upregulation of hippocampal *CNR1* expression (evidenced by real-time RT-qPCR) following sub-chronic administration of OS, HU-910, and IMI may reflect transcriptional changes consistent with a potential shared adaptive response to repeated treatment, although the functional significance of these changes remains to be determined. This is conceptually aligned with the broader literature showing that chronic antidepressant exposure can recruit ECS adaptations, including increased CB1 receptor density and signaling in stress- and mood-relevant circuits, which has been proposed to contribute to normalization of HPA-axis reactivity and affective behavior [51].

In the present study, *CNR1* upregulation occurred despite attenuation of most acute behavioral effects after repeated dosing. This is consistent with potential pharmacodynamic adaptation or tolerance following repeated cannabinoid receptor activation [52,53], although contributions from pharmacokinetic factors cannot be excluded. Still, our findings may suggest that ECS recruitment reflects transcriptional changes consistent with compensatory neuroplastic processes rather than immediate behavioral effects. The absence of changes in *FAAH* expression may further suggest receptor-level processes rather than altered endocannabinoid degradation. These transcriptional changes should, however, be interpreted cautiously, as mRNA changes do not necessarily translate into corresponding changes in CB1 protein expression or receptor function. Furthermore, the absence of such molecular adaptations in females may suggest that ECS transcriptional plasticity is differentially moderated by sex, although this observation may also reflect sex-dependent pharmacokinetic variability. While these findings may suggest transcriptional changes consistent with an adaptive response, this interpretation remains speculative in the absence of protein-level validation or functional assays.

### 3.3. OS: Sexually Dimorphic Responsiveness

The acute antidepressant-like effects of OS were limited to females, while anxiolytic-like effects showed a sex-dependent pattern. In females, acutely administered OS produced both antidepressant- and anxiolytic-like responses, while in males, only an acute anxiolytic-like effect was observed, evidenced by increased time and entries to the OFT center. Estrogen is known to modulate mood regulation [54,55] and endocannabinoid signaling [56,57], and fluctuations in estradiol influence affective behavior [55,58,59]. These findings may reflect pharmacodynamic mechanisms, such as the interaction of OS with estrogen-dependent regulatory pathways, amplifying affective responsiveness in females. This mechanism may contribute to the pronounced acute antidepressant- and anxiolytic-like effects observed in females, although pharmacokinetic differences between sexes may also play a role. Since female rats were of similar ages in both the acute and sub-chronic studies, the absence of sub-chronic behavioral effects in females may reflect pharmacodynamic adaptation or dose-dependent dynamics rather than a decline in estrogen. However, sex-dependent pharmacokinetic differences cannot be excluded.

In males, anxiolytic-like effects were observed following both acute and sub-chronic administration of OS, although the behavioral indices differed among the studies. The acute effect was reflected by increased center exploration in the OFT and suggests a reduction in anxiety-like behavior shortly after OS administration. The sub-chronic effect was reflected by reduced freezing duration in the OFT, which was accompanied by increased hippocampal *CNR1* expression. Since hippocampal CB1 signaling is widely implicated in regulating stress responses and emotional learning, the observed increase in *CNR1* expression following sub-chronic OS administration may reflect transcriptional changes in endocannabinoid signaling in the hippocampus, which could be associated with the observed behavioral improvements [60], although functional validation is required. In contrast to females, the delayed and primarily anxiolytic-like effects observed in males suggest that OS may preferentially engage slower, CB1-related neuroplastic processes in the absence of estrogen-driven modulation, although differences in pharmacokinetic profiles between sexes may also contribute. Future studies should evaluate multiple sub-chronic and chronic dosing regimens, including lower doses and intermittent administration schedules. This can help elucidate whether tolerance-like adaptations can be mitigated and behavioral efficacy can be improved.

### 3.4. HU-910: Temporal Divergence and Indirect ECS Recruitment in Males

HU-910 produced acute antidepressant-like effects in males that did not persist with sub-chronic dosing; instead, an anxiolytic-like trend emerged. This temporal divergence may reflect distinct phases of CB2-mediated action, although pharmacokinetic factors may also contribute to these time-dependent effects. Acutely, CB2 activation may rapidly modulate immune signaling or monoaminergic transmission, indirectly producing an immediate antidepressant-like response [33,61]. However, with prolonged exposure, receptor desensitization, altered intracellular signaling, or downstream neuroplastic processes, as shown with other CB2 agonists [62], antidepressant-like efficacy could diminish, alongside shifting effects towards anxiolysis. The observed sub-chronic upregulation of hippocampal *CNR1*, despite being undetectable in hippocampal *CNR2*, suggests indirect ECS recruitment, potentially via CB2-mediated immunomodulation, predominantly peripheral or microglial. Thus, HU-910 may influence inflammatory processes outside the hippocampus and secondarily affect hippocampal inflammatory tone and CB1 set-point. While these findings may support the hypothesis that CB2-mediated effects are indirectly shaped by sex-specific immune and hormonal environments, resulting in sexually dimorphic temporal and behavioral outcomes, differences in pharmacokinetic profiles between sexes may also contribute to these results. Nevertheless, our findings are consistent with evidence of CB2 agonists’ potent anti-inflammatory actions (e.g., HU-910 suppressing pro-inflammatory mediators in vivo) and the well-supported inflammation–mood link [42,43,63].

The limited behavioral and molecular responsiveness following sub-chronic administration in females may reflect sex-specific pharmacodynamic differences and/or pharmacokinetic variability [64], or hormonal modulation of endocannabinoid signaling [65]. Future work should investigate the pharmacokinetics of HU-910 in both sexes and test behavioral responses across estrous cycle stages to better interpret these interactions.

### 3.5. IMI: Sex-Differentiated Anxiogenic Effects

Our finding that IMI induced antidepressant-like effects in both sexes but failed to produce anxiolytic-like effects in either sex, with females instead showing anxiogenic-like responses (reduced entries to OFT center and elevated hair CORT levels), highlights well-established sex differences in antidepressant responsiveness [66,67,68]. However, given the ongoing discussion regarding the reliability of hair CORT as a biomarker of chronic stress [69], the elevated hair CORT levels in females should be interpreted cautiously and in conjunction with behavioral data. IMI‘s serotonergic and noradrenergic mechanisms [70] are differentially modulated by sex hormones, shaping antidepressant efficacy in both sexes. Specifically, testosterone has been shown to enhance noradrenergic signaling [71], while estrogen preferentially modulates serotonergic systems [72]. In addition, sex-dependent pharmacokinetic differences may contribute to these effects, as IMI metabolism is known to differ between males and females, potentially leading to differences in drug exposure and active metabolite formation [73]. Future studies assessing serotonin and noradrenaline-related activity alongside sex hormone levels may help elucidate these interactions.

### 3.6. SwissADME Predictions Based on Molecular Structures

To address the possibility that the sex-dependent behavioral effects observed in the present study were influenced not only by pharmacodynamics but also by pharmacokinetics, we performed an exploratory in silico ADME analysis using SwissADME. Because the compounds were administered intraperitoneally, oral absorption was not considered the key parameter; instead, the most relevant outputs were predicted BBB permeability, P-glycoprotein (P-gp) substrate liability, and cytochrome P450 (CYP) interaction profiles. This analysis indicated clear differences among the tested compounds. IMI was predicted to be BBB-permeant, whereas OS and HU-910 were predicted not to be BBB-permeant and to be P-gp substrates. HU-910 additionally showed extreme lipophilicity and very poor predicted solubility, suggesting potentially complex disposition. These predictions do not provide sex-specific PK data per se, since SwissADME is structure-based and therefore sex-independent, but they identify plausible pathways through which male–female differences in exposure could arise. This point is particularly relevant for IMI, whose disposition is already known to depend on CYP2C19-mediated demethylation to desipramine and CYP2D6-mediated hydroxylation, and for which sex-dependent pharmacokinetics have been demonstrated in rats, with females showing slower initial metabolism and higher desipramine levels than males [73]. Moreover, experimental studies indicate that P-gp can influence IMI transport across the BBB [74,75]. In contrast, for OS, the more plausible metabolic framework is not classical CYP metabolism but handling within the N-acyl amino acid/endocannabinoidome network, in which FAAH and PM20D1 act as complementary synthase/hydrolase pathways [74,76]. For HU-910, the present interpretation remains more exploratory, as its disposition pathways are less well characterized, and our inference is based mainly on its physicochemical profile and predicted transporter liability. Taken together, these considerations suggest that the sex-dependent behavioral responses observed in the current study should not be interpreted as evidence of purely sex-dependent pharmacodynamic sensitivity. Rather, they may reflect a combination of pharmacodynamic and pharmacokinetic factors, and direct plasma and brain exposure studies will be necessary to disentangle their relative contributions [73,75,76,77].

### 3.7. General Limitations and Future Directions

The following limitations should be considered. First, the less-pronounced sub-chronic effects, compared to acute behavioral effects, may be a result of some desensitization and tolerance. Future studies should assess alternative dosing regimens, including lower sub-chronic doses and intermittent schedules to mitigate tolerance (e.g., every other day, 2 times a week, etc.), and extended chronic three–four-week administration to determine optimal therapeutic windows. Second, strain-specific stress reactivity in WKY rats also limits generalizability, highlighting the need for replication in additional models. Third, the female estrous cycle stage was not systemically assessed. Given the likely possible interaction between sex hormones, pharmacological agents, and ECS signaling, future research should evaluate behavioral and molecular outcomes across different stages of the female estrous cycle. The relatively small sample size (N = 6/group) in the molecular analysis may also be a limitation, though the variability of real-time PCR is not so large in our experience.

An additional limitation is that ECS-related molecular changes were assessed only at the mRNA level by RT-qPCR. Thus, the observed increase in hippocampal CNR1 after sub-chronic treatment should be interpreted as evidence of transcriptional upregulation, not as direct evidence of increased CB1 receptor protein. Since the ECS regulation is not exclusively transcription-driven and may be substantially influenced by receptor trafficking, desensitization, protein stability, and enzyme activity, the present RT-qPCR data should be interpreted as transcriptional evidence only; confirmation at the protein and/or functional level via Western blot or immunohistochemistry will be required in future work. Finally, regarding hair CORT assessment, we note that the reliability of this biomarker has been challenged recently, particularly regarding temporal resolution and potential external contamination [69]. Thus, the hair CORT findings in the present study should be interpreted, with caution, as exploratory and in conjunction with behavioral outcomes rather than as a definitive measure of physiological stress.

Taken together, these findings suggest that sex differences in response to OS and HU-910 are not only behavioral but may also reflect distinct underlying biological mechanisms, including hormone-sensitive ECS modulation, differential neuroimmune signaling, and sex-specific recruitment of hippocampal plasticity. However, the relative contributions of pharmacodynamic and pharmacokinetic factors remain to be determined.

## 4. Materials and Methods

### 4.1. Animals

The acute study included 71 WKY 3.5–5-month-old male (N = 35) and female (N = 36) rats, with a mean weight of 294 g and 183 g, respectively. The sub-chronic study initially included 95 WKY rats, specifically 3–7-month-old male (N = 47) and 3.5–6-month-old female (N = 48) rats, with a mean weight of 289 g and 169 g, respectively. However, eight rats died during the sub-chronic study, four males from a higher dose of IMI (15 mg/kg i.p.) and two males and two females for unknown reasons. Despite the literature on the efficacy of chronic administration of 15 mg/kg i.p IMI in male WKY rats [78], we lowered the males’ sub-chronic IMI dose to 10 mg/kg i.p., which has been shown to produce antidepressant-like effects in male WKY rats [79]. The rats were housed in polysulfone cages (38 × 21 × 18 cm), with 2–3 same-sex rats per cage, fitted with a plastic tube for enrichment, in a temperature-controlled facility (22 ± 1 °C) under standard laboratory conditions. The cages facilitated access to food and water ad libitum, and we applied a 12 h light/dark cycle (lights on at 07:00 h). The WKY rats were sourced from Bar-Ilan University’s colony. The animal study protocol was approved by Bar-Ilan University’s Institutional Animal Care and Use Committee (protocol code IL-2312-130-4; date of approval: 14 December 2023) and complied with the guidelines of the Society for Neuroscience.

In a previous study conducted in our lab [22], the acute effect of orally consumed cannabidiolic acid methyl ester was assessed in the same strain and sex of the rats used in our proposed study, using the same FST paradigm. In that study, on the immobility measure, an effect size of 1.6 (*p* < 0.05) was found. Based on these data, a power analysis showed that the minimal number of subjects per group needed to find this effect size, with power = 80, is 8. Thus, in the current study’s acute and sub-chronic experiments, we used N = 10–12 per group to allow for potential subject attrition and/or rejection of outliers.

### 4.2. Drugs and Design

**Figure 4:**
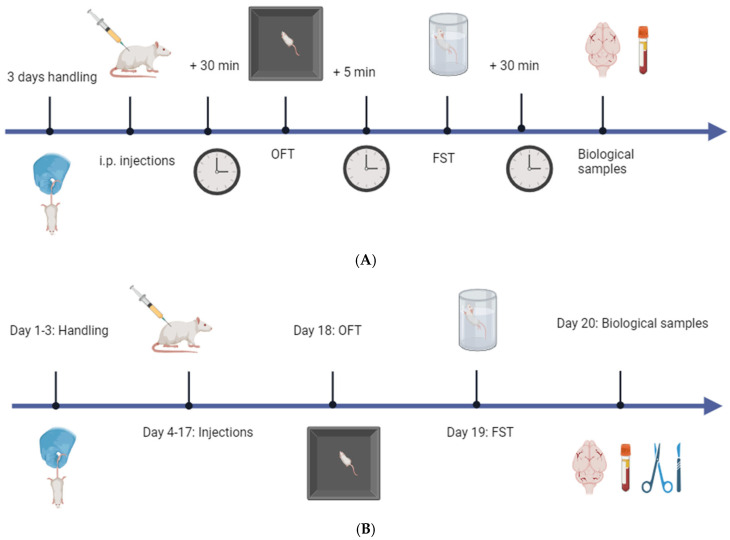
**Experimental design and behavioral testing timeline for the acute and sub-chronic studies.** (**A**) Acute administration schedule. Rats were handled for three days prior to testing, followed by a single i.p. injection of the tested compounds. Behavioral testing was conducted 30 min after injections using the OFT to assess anxiety-like behavior, followed 5 min later by the FST to assess depression-like behavior. Blood and brain tissues were collected 30 min after completion of behavioral testing for subsequent molecular and hormonal analyses but were not used in this study. (**B**) Sub-chronic treatment schedule. Rats were handled for three days and subsequently received daily i.p. injections of the tested compounds from day 4 to 17. Behavioral testing was conducted on day 18 (OFT) and day 19 (FST), followed by the collection of blood, brain tissue, and hair samples on day 20 for gene expression and endocrine analyses. Abbreviations: OFT—Open Field Test; FST—Forced Swim Test; i.p.—intraperitoneal. Created with BioRender.com.

OS and HU-910 were dissolved in a 1:1:18 ratio of ethanol:Tween 80:saline on the day of the study. The vehicle consisted of a solution of ethanol, Tween 80, and saline in a 1:1:18 ratio and was prepared one day before the study in the case of the acute administration study or on each day of the 14-day sub-chronic administration study. Imipramine Hydrochloride (Sigma Aldrich, Saint Louis, MO, USA) was dissolved in 3 parts ethanol to 1 part water, followed by a 1:1:18 ratio of ethanol:Tween 80:saline. The drugs and vehicle were injected i.p. in a 10 mL/kg volume 30 min before behavioral testing began in the acute administration study or each morning of the sub-chronic administration study. Animals received a dose of 7.5 mg/kg N-oleoyl-l-serine (OS) (N = 12 per sex, acute and sub-chronic), 5 mg/kg HU-910 (N = 11 males, acute and sub-chronic; N = 12 females, acute and sub-chronic), or vehicle (N = 12 per sex, acute and sub-chronic) acutely or daily for 14 days, excluding Saturdays. Additionally, in the sub-chronic study, animals received a dose of 10 mg/kg IMI (N = 12 per sex) daily for 14 days, excluding Saturdays. OS and HU-910 were synthesized as described previously [25,80,81,82]. In the case of the acute study, behavioral tests (OFT and FST) were performed thirty minutes after receiving the injections, with a five-minute break in between. In the case of the sub-chronic study, all rats underwent the OFT one day after the last injection. The following day, they underwent the FST. The next day, all rats were sacrificed, and trunk blood and brain tissue were collected for analysis of underlying biological mechanisms. Additionally, the rats were shaved post mortem to measure post-treatment corticosterone levels in the hair via Enzyme-Linked Immunosorbent Assay (ELISA). A schematic representation of the experimental design is provided in Figure 4.

### 4.3. Behavioral Tests

#### 4.3.1. Forced Swim Test

The FST was as described by Porsolt et al. [83], with modifications. A Plexiglas cylinder (45.5 cm tall, 20 cm diameter) was filled with 24 ± 0.5 °C water according to the animal’s weight and in a manner that prevented them from touching the bottom. The animals were immersed in the Plexiglas cylinder for 5 min, and their behavior was recorded using “Ethovision” software (Version 15). The measures taken were the duration of immobility (making only minimal movements to keep the head above water) and struggling (making active forepaw movements in and out of the water, including climbing). These measures were analyzed manually using “Solomon Coder” software (19.08.02). At completion of the test, the animals were dried off with a towel, the cylinder was cleaned, and the water was changed between test animals.

#### 4.3.2. Open Field Test (OFT)

The Open Field arena (65 cm × 65 cm) was enclosed by walls 40 cm high. The floor space was divided into nine equal-sized squares (3 × 3): four corners, four periphery areas, and the center for data analysis. The subject was gently placed in the center and allowed to explore the apparatus for 5 min. Thereafter, the animal was returned to the home cage, and the apparatus was cleaned with 70% ethanol. The animals’ behavior was videotaped and analyzed offline using “Ethovision” software. Two groups of measures were assessed via “Ethovision.” (1) Activity measures included distance traveled and mobility. (2) Anxiety/exploratory-like measures included number of entries to the center, time spent in the center, number of entries to the corners, and time spent in the corners. Freeze duration and frequency, as well as groom frequency, were manually analyzed using “Solomon Coder” software.

### 4.4. Steroid Extraction and ELISA—Hair Samples

Post sub-chronic study, male and female hair samples (n = 6–9 per group) were transferred to Petri dishes and washed twice with approximately 40 mL isopropanol for three minutes on a plate rotator at 100 rpm. Once dry, approximately 20 mg (based on a validation) of hair from each sample was weighed and placed in a glass vial, together with 3 mL methanol. The vials were then sonicated for 30 min, followed by incubation at 50 °C on a plate rotator at 160 rpm for 20 h. Thereafter, the methanol from each vial was transferred to 1.5 mL microcentrifuge tubes and centrifuged at 14,000 rpm at 4 °C for 10 min. Supernatants were transferred into glass tubes, dried in a sample concentrator under a stream of nitrogen, and refrigerated at 4 °C until the ELISA. We quantified hair corticosterone levels using a corticosterone ELISA kit ADI-900-097 following the manufacturer’s instructions (Enzo Life Sciences, Farmingdale, NY, USA) and a previous protocol [84]. Each sample was reconstituted with methanol (22 μL) and assay buffer (218 μL) and dispensed into the appropriate wells according to the manufacturer’s instructions. Absorbance at each assay was read at a wavelength of 405 and 590 nm with a plate reader (BioTek^®^ 800 TS absorbance reader, Agilent Technologies, Santa Clara, CA, USA). Based on standard curves run in duplicate on each plate, CORT concentration was determined with the aid of Gen5 software (version 3.14). The manufacturer reported that cross-reactivity for the CORT kit was 28.6% for deoxycorticosterone, 1.7% for progesterone, and less than 0.28% for all other steroids.

Kits were validated for rat hair by showing the parallelism between hair CORT levels and the standards that were provided with the kit (*p* = 0.688). The linear range for CORT was between 10 and 35 mg of hair (equivalent to 160 pg/mL and 800 pg/mL standard; R^2^ = 0.956, *p* < 0.0001). The average inter-assay coefficient of variance (CV) was 7.3% for three repeats (mean concentration ± standard deviation [SD]: 1749.704 ± 127.719). The average intra-assay CV was 15.17% for six repeats (mean concentration ± SD: 1748.45 ± 265.166). Recovery was 82.17%.

### 4.5. Quantitative RT-PCR Analysis

Total RNA was isolated from hippocampal tissue (n = 6 per group) obtained from male and female WKY rats (four experimental groups: vehicle, HU-910, OS, IMI) utilizing the RNeasy Lipid Tissue Mini Kit (Qiagen, Singapore, Cat. no. 74804) according to the manufacturer’s instructions. RNA was eluted in 30 μL RNase-free water, and RNA concentration was determined using a NanoDrop One Microvolume UV-Vis Spectrophotometer (Thermo Fisher Scientific, Waltham, MA, USA). cDNA was synthesized from total RNA using the High-Capacity cDNA Reverse Transcription Kit (Applied Biosystems™, Foster City, CA, USA, Cat. no. 4374966, Thermo Fisher Scientific) following the manufacturer’s guidelines. Gene expression levels of *CNR1* (Assay ID: Rn.PT.58.46210215, ref sequence NM_012784), *CNR2* (Assay ID: Rn.PT. 58.18327829, ref sequence NM_020543), and *GAPDH* (Assay ID: Rn.PT.58.35727291, ref sequence NM_017008) were quantified using predesigned PrimeTime qPCR Primer Assays (Integrated DNA Technologies, Coralville, IA, USA). Primers, as well as a probe, targeting *FAAH* were designed specifically for this study, with the following sequences: probe: 5′-/56-FAM/CCTGGGAAG/ZEN/TGAACAAAGGGACCAAC/3IABKFQ/-3′; forward primer: 5′-CAGGGACACCATAGAGCAG-3′; reverse primer: 5′-AGAGGCTGTGTTCTTTACTTACC-3′.

All qPCR reactions utilized Prime-Time qPCR Primer Assays (Integrated DNA Technologies, Coralville, IA, USA), incorporating the FAM/ZEN/IBFQ double-quenched probe configuration. *GAPDH* served as the endogenous reference gene. RT-PCR was performed on an Azure Cielo Real-Time PCR system (Azure Biosystems, Dublin, CA, USA). Each 20 μL reaction contained 3 μL cDNA, 10 μL of 2× master mix buffer, 1 μL Prime-Time qPCR Probe assay mix, and 6 μL of water. The thermal cycling conditions included an initial denaturation step at 95 °C for 3 min, followed by 40 cycles of 95 °C for 10 s and 60 °C for 30 s.

### 4.6. Statistical Analysis

Statistical analyses were performed using GraphPad Prism (version 10.1). Outlier detection was conducted using the ROUT method (Q = 5%). Group comparisons were conducted using one-way ANOVA, followed by Bonferroni test for post hoc analysis. In cases where the assumption of equal variances was violated, as determined by the Brown–Forsythe test, a Brown–Forsythe ANOVA was applied, followed by Dunnett T3 correction for multiple comparisons. In cases where the distribution was not normal, a Kruskal–Wallis ANOVA was applied, followed by Dunn’s correction for multiple comparisons. Data are presented as mean ± SEM. Statistical significance is indicated as follows: * *p* < 0.05, ** *p* < 0.01, *** *p* < 0.001, and **** *p* < 0.0001.

### 4.7. In Silico ADME Profiling

To explore whether compound-specific pharmacokinetic properties might plausibly contribute to the sex-dependent behavioral effects, the structures of OS, HU-910, and IMI were analyzed using SwissADME with default settings [77]. Predicted BBB permeability, P-glycoprotein substrate status, and CYP1A2, CYP2C19, CYP2C9, CYP2D6, and CYP3A4 interaction liabilities were extracted. These analyses were used for exploratory and hypothesis-generating purposes only and were not intended to substitute for experimental pharmacokinetic measurements.

## 5. Conclusions

In sum, OS and HU-910 demonstrate sex-dependent antidepressant- and anxiolytic-like effects, with acute behavioral responses generally stronger than sub-chronic responses. Further, sub-chronic administration of all compounds upregulated hippocampal CNR1 expression, which may be consistent with CB1-related plasticity at the transcriptional level, although functional validation is required to confirm this interpretation. The absence of significant gene expression changes in females may reflect sex-dependent variability or hormone-related buffering of ECS transcription, consistent with the complex sex-by-treatment interactions observed in the behavioral findings of our study.

These results emphasize the importance of considering sex as a biological variable and support the presence of sexually dimorphic treatment responses. Given the predominance of male-focused animal studies [85], despite depression and anxiety being approximately twice as prevalent in females [6,7,8,9], our findings contribute to a better understanding of the putative sex differences in response to both conventional antidepressants (e.g., IMI) and emerging pharmacological agents (e.g., OS, HU-910). One should keep in mind, though, that the present findings demonstrate sex-dependent behavioral responses to OS, HU-910, and IMI, but the current dataset does not allow these differences to be assigned exclusively to pharmacodynamic mechanisms, since sex-dependent variation in metabolism, transporter activity, and brain exposure may also contribute substantially [73,74,75,76,86].

Furthermore, the convergent increase in hippocampal CNR1 expression suggests that ECS-related transcriptional changes may represent a shared pathway across treatments, although further protein-level and functional assays are required to confirm their adaptive significance. Future research using combination paradigms with classical antidepressants may help determine whether these CB1-related transcriptional changes translate into more robust or faster-onset antidepressant and anxiolytic effects, with a potentially improved side effect profile relative to tricyclics. As the endocannabinoid system continues to gain attention for its role in depression and anxiety [87,88], these findings contribute to advancing more mechanistically informed and sex-sensitive therapeutic treatments.

## Figures and Tables

**Table 1:** Predicted SwissADME descriptors for HU-910, oleoyl serine, and IMI exported from the SwissADME CSV output. Values include physicochemical properties, solubility, pharmacokinetic parameters, drug-likeness filters, and medicinal chemistry alerts. Because these predictions are structure-based, they are not sex-specific, and they were used only for exploratory interpretation of potential disposition-related differences among compounds. Abbreviations: SMILES, simplified molecular input line entry system; MW, molecular weight; TPSA, topological polar surface area; P-gp, P-glycoprotein; BBB, blood–brain barrier; GI, gastrointestinal; CYP, cytochrome P450.

Molecule	Canonical SMILES	Formula	MW	TPSA	Consensus Log P	GI Absorption	BBB Permeant	P-gp Substrate	CYP1A2 Inhibitor	CYP2C19 Inhibitor	CYP2C9 Inhibitor	CYP2D6 Inhibitor	CYP3A4 Inhibitor
HU-910	CCCCCCC(C)(C)C1=CC(OC)=C(C2=CC3CCC2(CO)C3(C)C)C(OC)=C1	C_27_H_42_O_3_	414.62	38.69	6.34	Low	No	Yes	No	No	No	Yes	No
Oleoyl Serine	CCCCCCCC/C=C\CCCCCCCC(=O)N[C@H](C(=O)O)CO	C_21_H_39_NO_4_	369.54	86.63	4.48	High	No	Yes	Yes	Yes	Yes	Yes	No
Imipramine	CN(CCCN1c2ccccc2CCc2c1cccc2)C	C_19_H_24_N_2_	280.41	6.48	3.8	High	Yes	No	Yes	No	No	Yes	No

## Data Availability

The raw data supporting the conclusions of this article will be made available by the authors on request.

## References

[1] Otte C., Gold S.M., Penninx B.W., Pariante C.M., Etkin A., Fava M., Mohr D.C., Schatzberg A.F. (2016). Major Depressive Disorder. Nat. Rev. Dis. Primers.

[2] Machmutow K., Meister R., Jansen A., Kriston L., Watzke B., Härter M.C., Liebherz S. (2019). Comparative Effectiveness of Continuation and Maintenance Treatments for Persistent Depressive Disorder in Adults. Cochrane Database Syst. Rev..

[3] Fornaro M., Anastasia A., Novello S., Fusco A., Pariano R., De Berardis D., Solmi M., Veronese N., Stubbs B., Vieta E. (2019). The Emergence of Loss of Efficacy during Antidepressant Drug Treatment for Major Depressive Disorder: An Integrative Review of Evidence, Mechanisms, and Clinical Implications. Pharmacol. Res..

[4] Pigott H.E., Leventhal A.M., Alter G.S., Boren J.J. (2010). Efficacy and Effectiveness of Antidepressants: Current Status of Research. Psychother. Psychosom..

[5] Cipriani A., Furukawa T.A., Salanti G., Chaimani A., Atkinson L.Z., Ogawa Y., Leucht S., Ruhe H.G., Turner E.H., Higgins J.P.T. (2018). Comparative Efficacy and Acceptability of 21 Antidepressant Drugs for the Acute Treatment of Adults with Major Depressive Disorder: A Systematic Review and Network Meta-Analysis. Lancet.

[6] McLean C.P., Asnaani A., Litz B.T., Hofmann S.G. (2011). Gender Differences in Anxiety Disorders: Prevalence, Course of Illness, Comorbidity and Burden of Illness. J. Psychiatr. Res..

[7] Salk R.H., Hyde J.S., Abramson L.Y. (2017). Gender Differences in Depression in Representative National Samples: Meta-Analyses of Diagnoses and Symptoms. Psychol. Bull..

[8] Pavlidi P., Kokras N., Dalla C. (2022). Sex Differences in Depression and Anxiety. Sex Differences in Brain Function and Dysfunction.

[9] Van de Velde S., Bracke P., Levecque K. (2010). Gender Differences in Depression in 23 European Countries. Cross-National Variation in the Gender Gap in Depression. Soc. Sci. Med..

[10] Hill M.N., Gorzalka B.B. (2005). Is There a Role for the Endocannabinoid System in the Etiology and Treatment of Melancholic Depression?. Behav. Pharmacol..

[11] Mangieri R.A., Piomelli D. (2007). Enhancement of Endocannabinoid Signaling and the Pharmacotherapy of Depression. Pharmacol. Res..

[12] Steiner M.A., Marsicano G., Nestler E.J., Holsboer F., Lutz B., Wotjak C.T. (2008). Antidepressant-like Behavioral Effects of Impaired Cannabinoid Receptor Type 1 Signaling Coincide with Exaggerated Corticosterone Secretion in Mice. Psychoneuroendocrinology.

[13] Vinod K.Y., Hungund B.L. (2006). Role of the Endocannabinoid System in Depression and Suicide. Trends Pharmacol. Sci..

[14] Hill M.N., Hillard C.J., Bambico F.R., Patel S., Gorzalka B.B., Gobbi G. (2009). The Therapeutic Potential of the Endocannabinoid System for the Development of a Novel Class of Antidepressants. Trends Pharmacol. Sci..

[15] Sarris J., Sinclair J., Karamacoska D., Davidson M., Firth J. (2020). Medicinal Cannabis for Psychiatric Disorders: A Clinically-Focused Systematic Review. BMC Psychiatry.

[16] Dlugos A., Childs E., Stuhr K.L., Hillard C.J., de Wit H. (2012). Acute Stress Increases Circulating Anandamide and Other N-Acylethanolamines in Healthy Humans. Neuropsychopharmacology.

[17] Redlich C., Dlugos A., Hill M.N., Patel S., Korn D., Enneking V., Foerster K., Arolt V., Domschke K., Dannlowski U. (2021). The Endocannabinoid System in Humans: Significant Associations between Anandamide, Brain Function during Reward Feedback and a Personality Measure of Reward Dependence. Neuropsychopharmacology.

[18] Wei D., Lee D., Li D., Daglian J., Jung K.-M., Piomelli D. (2016). A Role for the Endocannabinoid 2-Arachidonoyl-Sn-Glycerol for Social and High-Fat Food Reward in Male Mice. Psychopharmacology.

[19] Manduca A., Morena M., Campolongo P., Servadio M., Palmery M., Trabace L., Hill M.N., Vanderschuren L.J.M.J., Cuomo V., Trezza V. (2015). Distinct Roles of the Endocannabinoids Anandamide and 2-Arachidonoylglycerol in Social Behavior and Emotionality at Different Developmental Ages in Rats. Eur. Neuropsychopharmacol..

[20] Lanz C., Mattsson J., Stickel F., Dufour J.-F., Brenneisen R. (2018). Determination of the Endocannabinoids Anandamide and 2-Arachidonoyl Glycerol with Gas Chromatography-Mass Spectrometry: Analytical and Preanalytical Challenges and Pitfalls. Med. Cannabis Cannabinoids.

[21] Rouzer C.A., Ghebreselasie K., Marnett L.J. (2002). Chemical Stability of 2-Arachidonylglycerol under Biological Conditions. Chem. Phys. Lipids.

[22] Hen-Shoval D., Amar S., Shbiro L., Smoum R., Haj C.G., Mechoulam R., Zalsman G., Weller A., Shoval G. (2018). Acute Oral Cannabidiolic Acid Methyl Ester Reduces Depression-like Behavior in Two Genetic Animal Models of Depression. Behav. Brain Res..

[23] Hen-Shoval D., Moshe L., Indig-Naimer T., Mechoulam R., Shoval G., Zalsman G., Kogan N.M., Weller A. (2023). Cannabinoid Receptor 2 Blockade Prevents Anti-Depressive-like Effect of Cannabidiol Acid Methyl Ester in Female WKY Rats. Int. J. Mol. Sci..

[24] Hen-Shoval D., Indig-Naimer T., Moshe L., Kogan N.M., Zaidan H., Gaisler-Salomon I., Okun E., Mechoulam R., Shoval G., Zalsman G. (2024). Unraveling the Molecular Basis of Cannabidiolic Acid Methyl Ester’s Anti-Depressive Effects in a Rat Model of Treatment-Resistant Depression. J. Psychiatr. Res..

[25] Smoum R., Bar A., Tan B., Milman G., Attar-Namdar M., Ofek O., Stuart J.M., Bajayo A., Tam J., Kram V. (2010). Oleoyl Serine, an Endogenous *N*-Acyl Amide, Modulates Bone Remodeling and Mass. Proc. Natl. Acad. Sci. USA.

[26] Jin P., Yu H.-L., Lan T., Zhang F., Quan Z.-S. (2015). Antidepressant-like Effects of Oleoylethanolamide in a Mouse Model of Chronic Unpredictable Mild Stress. Pharmacol. Biochem. Behav..

[27] Piscitelli F., Guida F., Luongo L., Iannotti F.A., Boccella S., Verde R., Lauritano A., Imperatore R., Smoum R., Cristino L. (2020). Protective Effects of *N*-Oleoylglycine in a Mouse Model of Mild Traumatic Brain Injury. ACS Chem. Neurosci..

[28] Cortez I.L., Silva N.R., Rodrigues N.S., Pedrazzi J.F.C., Del Bel E.A., Mechoulam R., Gomes F.V., Guimarães F.S. (2022). HU-910, a CB2 Receptor Agonist, Reverses Behavioral Changes in Pharmacological Rodent Models for Schizophrenia. Prog. Neuropsychopharmacol. Biol. Psychiatry.

[29] Ashton J.C., Friberg D., Darlington C.L., Smith P.F. (2006). Expression of the Cannabinoid CB2 Receptor in the Rat Cerebellum: An Immunohistochemical Study. Neurosci. Lett..

[30] Onaivi E.S., Ishiguro H., Gong J.-P., Patel S., Meozzi P.A., Myers L., Perchuk A., Mora Z., Tagliaferro P.A., Gardner E. (2008). Brain Neuronal CB2 Cannabinoid Receptors in Drug Abuse and Depression: From Mice to Human Subjects. PLoS ONE.

[31] Van Sickle M.D., Duncan M., Kingsley P.J., Mouihate A., Urbani P., Mackie K., Stella N., Makriyannis A., Piomelli D., Davison J.S. (2005). Identification and Functional Characterization of Brainstem Cannabinoid CB_2_ Receptors. Science.

[32] Magid L., Heymann S., Elgali M., Avram L., Cohen Y., Liraz-Zaltsman S., Mechoulam R., Shohami E. (2019). Role of CB2 Receptor in the Recovery of Mice after Traumatic Brain Injury. J. Neurotrauma.

[33] Bauer M.E., Teixeira A.L. (2019). Inflammation in Psychiatric Disorders: What Comes First?. Ann. N. Y. Acad. Sci..

[34] Duivis H.E., Vogelzangs N., Kupper N., De Jonge P., Penninx B.W.J.H. (2013). Differential Association of Somatic and Cognitive Symptoms of Depression and Anxiety with Inflammation: Findings from the Netherlands Study of Depression and Anxiety (NESDA). Psychoneuroendocrinology.

[35] Lamers F., Vogelzangs N., Merikangas K.R., De Jonge P., Beekman A.T.F., Penninx B.W.J.H. (2013). Evidence for a Differential Role of HPA-Axis Function, Inflammation and Metabolic Syndrome in Melancholic versus Atypical Depression. Mol. Psychiatry.

[36] O’Donovan A., Hughes B.M., Slavich G.M., Lynch L., Cronin M.T., O’Farrelly C., Malone K.M. (2010). Clinical Anxiety, Cortisol and Interleukin-6: Evidence for Specificity in Emotion-Biology Relationships. Brain Behav. Immun..

[37] Pitsavos C., Panagiotakos D.B., Papageorgiou C., Tsetsekou E., Soldatos C., Stefanadis C. (2006). Anxiety in Relation to Inflammation and Coagulation Markers, among Healthy Adults: The ATTICA Study. Atherosclerosis.

[38] Amenta P.S., Jallo J.I., Tuma R.F., Elliott M.B. (2012). A Cannabinoid Type 2 Receptor Agonist Attenuates Blood–Brain Barrier Damage and Neurodegeneration in a Murine Model of Traumatic Brain Injury. J. Neurosci. Res..

[39] Kasatkina L.A., Rittchen S., Sturm E.M. (2021). Neuroprotective and Immunomodulatory Action of the Endocannabinoid System under Neuroinflammation. Int. J. Mol. Sci..

[40] Morcuende A., García-Gutiérrez M.S., Tambaro S., Nieto E., Manzanares J., Femenia T. (2022). Immunomodulatory Role of CB2 Receptors in Emotional and Cognitive Disorders. Front. Psychiatry.

[41] Tanaka M., Sackett S., Zhang Y. (2020). Endocannabinoid Modulation of Microglial Phenotypes in Neuropathology. Front. Neurol..

[42] Chistyakov D.V., Astakhova A.A., Sergeeva M.G. (2018). Resolution of Inflammation and Mood Disorders. Exp. Mol. Pathol..

[43] Jones B.D.M., Daskalakis Z.J., Carvalho A.F., Strawbridge R., Young A.H., Mulsant B.H., Husain M.I. (2020). Inflammation as a Treatment Target in Mood Disorders: Review. BJPsych Open.

[44] Nam H., Clinton S.M., Jackson N.L., Kerman I.A. (2014). Learned Helplessness and Social Avoidance in the Wistar-Kyoto Rat. Front. Behav. Neurosci..

[45] Redei E.E., Udell M.E., Solberg Woods L.C., Chen H. (2023). The Wistar Kyoto Rat: A Model of Depression Traits. Curr. Neuropharmacol..

[46] Virijevic K., Spasojevic N., Stefanovic B., Ferizovic H., Jankovic M., Vasiljevic P., Dronjak S. (2024). Chronic Mild Stress-Induced Dysregulation of MAPK and PI3K/AKT Signaling in the Hippocampus and Medial Prefrontal Cortex of WKY Female Rats. Neurosci. Lett..

[47] Loizeau V., Durieux L., Mendoza J., Wiborg O., Barbelivien A., Lecourtier L. (2024). Behavioural Characteristics and Sex Differences of a Treatment-Resistant Depression Model: Chronic Mild Stress in the Wistar-Kyoto Rat. Behav. Brain Res..

[48] Papp M., Willner P. (2023). Models of Affective Illness: Chronic Mild Stress in the Rat. Curr. Protoc..

[49] Jennings E.M., Okine B.N., Olango W.M., Roche M., Finn D.P. (2016). Repeated Forced Swim Stress Differentially Affects Formalin-Evoked Nociceptive Behaviour and the Endocannabinoid System in Stress Normo-Responsive and Stress Hyper-Responsive Rat Strains. Prog. Neuropsychopharmacol. Biol. Psychiatry.

[50] Millard S.J., Weston-Green K., Newell K.A. (2020). The Wistar-Kyoto Rat Model of Endogenous Depression: A Tool for Exploring Treatment Resistance with an Urgent Need to Focus on Sex Differences. Prog. Neuropsychopharmacol. Biol. Psychiatry.

[51] Hill M.N., Ho W.-S.V., Sinopoli K.J., Viau V., Hillard C.J., Gorzalka B.B. (2006). Involvement of the Endocannabinoid System in the Ability of Long-Term Tricyclic Antidepressant Treatment to Suppress Stress-Induced Activation of the Hypothalamic–Pituitary–Adrenal Axis. Neuropsychopharmacology.

[52] Nguyen P.T., Schmid C.L., Raehal K.M., Selley D.E., Bohn L.M., Sim-Selley L.J. (2012). β-Arrestin2 Regulates Cannabinoid CB1 Receptor Signaling and Adaptation in a Central Nervous System Region–Dependent Manner. Biol. Psychiatry.

[53] Sim-Selley L.J. (2003). Regulation of Cannabinoid CB1 Receptors in the Central Nervous System by Chronic Cannabinoids. Crit. Rev. Neurobiol..

[54] Ji M., Yu Q. (2015). Primary Osteoporosis in Postmenopausal Women. Chronic Dis. Transl. Med..

[55] Soares C.N. (2013). Depression in Peri- and Postmenopausal Women: Prevalence, Pathophysiology and Pharmacological Management. Drugs Aging.

[56] Peterson B.M., Martinez L.A., Meisel R.L., Mermelstein P.G. (2016). Estradiol Impacts the Endocannabinoid System in Female Rats to Influence Behavioral and Structural Responses to Cocaine. Neuropharmacology.

[57] Santoro A., Mele E., Marino M., Viggiano A., Nori S.L., Meccariello R. (2021). The Complex Interplay between Endocannabinoid System and the Estrogen System in Central Nervous System and Periphery. Int. J. Mol. Sci..

[58] D’Souza D., Sadananda M. (2017). Estrous Cycle Phase-Dependent Changes in Anxiety-and Depression-like Profiles in the Late Adolescent Wistar-Kyoto Rat. Ann. Neurosci..

[59] Rachman I.M., Unnerstall J.R., Pfaff D.W., Cohen R.S. (1998). Estrogen Alters Behavior and Forebrain C-Fos Expression in Ovariectomized Rats Subjected to the Forced Swim Test. Proc. Natl. Acad. Sci. USA.

[60] Hillard C.J. (2014). Stress Regulates Endocannabinoid-CB1 Receptor Signaling. Semin. Immunol..

[61] Youssef D.A., El-Fayoumi H.M., Mahmoud M.F. (2019). Beta-Caryophyllene Alleviates Diet-Induced Neurobehavioral Changes in Rats: The Role of CB2 and PPAR-γ Receptors. Biomed. Pharmacother..

[62] Spyridakos D., Papadogkonaki S., Dionysopoulou S., Mastrodimou N., Polioudaki H., Thermos K. (2021). Effect of Acute and Subchronic Administration of (R)-WIN55,212-2 Induced Neuroprotection and Anti Inflammatory Actions in Rat Retina: CB1 and CB2 Receptor Involvement. Neurochem. Int..

[63] Horváth B., Magid L., Mukhopadhyay P., Bátkai S., Rajesh M., Park O., Tanchian G., Gao R.Y., Goodfellow C.E., Glass M. (2012). A New Cannabinoid CB _2_ Receptor Agonist HU-910 Attenuates Oxidative Stress, Inflammation and Cell Death Associated with Hepatic Ischaemia/Reperfusion Injury. Br. J. Pharmacol..

[64] Tham M., Yilmaz O., Alaverdashvili M., Kelly M.E.M., Denovan-Wright E.M., Laprairie R.B. (2019). Allosteric and Orthosteric Pharmacology of Cannabidiol and Cannabidiol-dimethylheptyl at the Type 1 and Type 2 Cannabinoid Receptors. Br. J. Pharmacol..

[65] Matheson J., Bourgault Z., Le Foll B. (2022). Sex Differences in the Neuropsychiatric Effects and Pharmacokinetics of Cannabidiol: A Scoping Review. Biomolecules.

[66] Baca E., Garcia-Garcia M., Porras-Chavarino A. (2004). Gender Differences in Treatment Response to Sertraline versus Imipramine in Patients with Nonmelancholic Depressive Disorders. Prog. Neuropsychopharmacol. Biol. Psychiatry.

[67] Kornstein S.G., Schatzberg A.F., Thase M.E., Yonkers K.A., McCullough J.P., Keitner G.I., Gelenberg A.J., Davis S.M., Harrison W.M., Keller M.B. (2000). Gender Differences in Treatment Response to Sertraline Versus Imipramine in Chronic Depression. Am. J. Psychiatry.

[68] Sloan D.M.E., Kornstein S.G. (2003). Gender Differences in Depression and Response to Antidepressant Treatment. Psychiatr. Clin. N. Am..

[69] Colding-Jørgensen P., Hestehave S., Abelson K.S.P., Kalliokoski O. (2023). Hair Glucocorticoids Are Not a Historical Marker of Stress—Exploring the Time-Scale of Corticosterone Incorporation into Hairs in a Rat Model. Gen. Comp. Endocrinol..

[70] Humble M. (2000). Noradrenaline and Serotonin Reuptake Inhibition as Clinical Principles: A Review of Antidepressant Efficacy. Acta Psychiatr. Scand..

[71] Carrier N., Kabbaj M. (2012). Testosterone and Imipramine Have Antidepressant Effects in Socially Isolated Male but Not Female Rats. Horm. Behav..

[72] Pitychoutis P.M., Pallis E.G., Mikail H.G., Papadopoulou-Daifoti Z. (2011). Individual Differences in Novelty-Seeking Predict Differential Responses to Chronic Antidepressant Treatment through Sex- and Phenotype-Dependent Neurochemical Signatures. Behav. Brain Res..

[73] Wilson M.A., Roy E.J. (1986). Pharmacokinetics of Imipramine Are Affected by Age and Sex in Rats. Life Sci..

[74] Dalla C., Pavlidi P., Sakelliadou D.-G., Grammatikopoulou T., Kokras N. (2022). Sex Differences in Blood–Brain Barrier Transport of Psychotropic Drugs. Front. Behav. Neurosci..

[75] O’Brien F., Clarke G., Fitzgerald P., Dinan T., Griffin B., Cryan J. (2012). Inhibition of P-glycoprotein Enhances Transport of Imipramine across the Blood–Brain Barrier: Microdialysis Studies in Conscious Freely Moving Rats. Br. J. Pharmacol..

[76] Kim J.T., Terrell S.M., Li V.L., Wei W., Fischer C.R., Long J.Z. (2020). Cooperative Enzymatic Control of N-Acyl Amino Acids by PM20D1 and FAAH. eLife.

[77] Daina A., Michielin O., Zoete V. (2017). SwissADME: A Free Web Tool to Evaluate Pharmacokinetics, Drug-Likeness and Medicinal Chemistry Friendliness of Small Molecules. Sci. Rep..

[78] Lahmame A., Del Arco C., Pazos A., Yritia M., Armario A. (1997). Are Wistar-Kyoto Rats a Genetic Animal Model of Depression Resistant to Antidepressants?. Eur. J. Pharmacol..

[79] Nagasawa M., Otsuka T., Yasuo S., Furuse M. (2015). Chronic Imipramine Treatment Differentially Alters the Brain and Plasma Amino Acid Metabolism in Wistar and Wistar Kyoto Rats. Eur. J. Pharmacol..

[80] Mechoulam R., Magid L., Shohami E., Bab I. (2010). Arylated Camphenes, Processes for Their Preparation and Uses Thereof.

[81] Magid L. (2012). Development and Pharmacological Evaluation of Novel CB2 Receptor Selective Agonists as Anti-Inflammatory and Neuroprotective Agents. Ph.D. Dissertation.

[82] Mechoulam R., Magid L., Shohami E., Bab I. (2014). Arylated Camphenes, Processes for Their Preparation and Uses Thereof.

[83] Porsolt R.D., Bertin A., Jalfre M. (1977). Behavioral Despair in Mice: A Primary Screening Test for Antidepressants. Arch. Int. Pharmacodyn. Ther..

[84] Arnon L., Hazut N., Tabachnik T., Weller A., Koren L. (2016). Maternal Testosterone and Reproductive Outcome in a Rat Model of Obesity. Theriogenology.

[85] Beery A.K., Zucker I. (2011). Sex Bias in Neuroscience and Biomedical Research. Neurosci. Biobehav. Rev..

[86] Waxman D.J., Holloway M.G. (2009). Sex Differences in the Expression of Hepatic Drug Metabolizing Enzymes. Mol. Pharmacol..

[87] Keller J., Gomez R., Williams G., Lembke A., Lazzeroni L., Murphy G.M., Schatzberg A.F. (2017). HPA Axis in Major Depression: Cortisol, Clinical Symptomatology and Genetic Variation Predict Cognition. Mol. Psychiatry.

[88] Maldonado R., Cabañero D., Martín-García E. (2020). The Endocannabinoid System in Modulating Fear, Anxiety, and Stress. Dialogues Clin. Neurosci..

